# The utility of caffeine citrate as a neuroprotectant in the early life of premature newborns: a literature review of the effects on neurodevelopmental outcomes

**DOI:** 10.3389/fped.2025.1682903

**Published:** 2025-12-18

**Authors:** Lauren Phung, Kaithlyn Duong, Rawad Obeid

**Affiliations:** 1 Oakland University William Beaumont School of Medicine, Rochester, MI, United States; 2 Department of Pediatrics, Division of Pediatric Neurology, Corewell Health Children’s Hospital, Royal Oak, MI, United States

**Keywords:** caffeine, preterm infants, brain magnetic resonance imaging, neuroprotection, ultrasound

## Abstract

The incidence of prematurity-related complications has decreased due to advancements in medical preventive and supportive measures, but the rate of neurodevelopmental impairment secondary to prematurity continues to increase. Due to the high metabolic demand of the central nervous system during early development and the age-related sensitivity of the cerebral white matter, perinatal intermittent hypoxia can lead to significant cerebral pathology persisting into adulthood. Caffeine citrate is one of the mainstay medical treatments for apnea of prematurity and is widely used in many neonatal intensive care units. Caffeine citrate's benefits include reducing time on mechanical ventilation, enhancing extubation success, and decreasing the incidence of bronchopulmonary dysplasia. There is also mounting evidence that caffeine citrate benefits neurodevelopmental outcomes, attributable to its positive effect on respiratory status and other mechanisms. Research has shown that caffeine citrate exerts an anti-inflammatory effect via the antagonism of adenosine receptors, reduces the production of reactive oxygen species, and supports the plasticity of the central nervous system. This article aims to review the most up-to-date evidence on caffeine citrate's effects on neuroprotection and its role in reducing the severity of neurodevelopmental impairment associated with prematurity.

## Introduction

The methylxanthine derivatives, like caffeine citrate (CC), have been known historically for their medicinal use, especially as stimulants for the central nervous system. One of their common uses in pediatrics is for apnea of prematurity. Apnea of prematurity is defined as an abrupt cessation of respiration lasting for 20 s in infants with a gestational age (GA) of less than 37 weeks (1). An estimated 75,000 singleton births are preterm. Currently, CC is widely used as a standard of care for the treatment and prevention of apnea of prematurity in the neonatal intensive care unit (NICU). However, CC's benefits were found to extend beyond reducing the incidence of apnea to improving neurodevelopmental outcomes (2).

Due to its structure, CC also serves as a non-selective antagonist of G-protein-coupled adenosine receptors such as adenosine receptors A1 (A1R) and A2A (A2AR), which are highly concentrated in the central nervous system and are implicated in neuronal inflammation (3). The overactivation of A2AR is associated with synaptic cytotoxicity upon acute brain injuries (4). CC has also been found to increase luminal calcium (Ca+) release by lowering the threshold of activation of ryanodine receptor (RyR)-mediated calcium channels (5). CC also exerts an antioxidant activity against damage from reactive oxygen species (ROS). By reducing oxidative stress and modulating inflammation, CC may prevent long-term white matter damage from hypoxia in preterm infants. CC has been shown to exert neuroprotective benefits via an anti-inflammatory effect, promoting myelination, and supporting vascular integrity in animal studies (Table 1). The current recommended loading dose and maintenance dose of CC intravenous administration for preterm infants by the U.S. Food and Drug Administration are 20 mg/kg and 5 mg/kg, respectively (12).

**Table 1:** Neuroprotective effects of caffeine in animal studies.

Study	Timing and type of injury	Treatment administration	Results
(6)	HI at P10	Single dose of caffeine or PBS given directly after HI	Caffeine administration post HI caused reduction in CD8+ T-lymphocytes activation.
(7)	HI at P6 and P7	P6 group -caffeine or saline given directly post HI and another dose caffeine or saline at 24 h later P7 group – single dose of caffeine given directly post HI	Caffeine treated HI P6 and P7 rats performed significantly better in Rotarod, non-spatial water maze, and silent gap discrimination task relative to the untreated HI group.
(8)	HI at P6	1st dose of caffeine or saline directly post HI and 2nd dose of caffeine or saline at 24 h post HI	Caffeine treated group had higher performance score in rotarod task, silent gap discrimination task, and non-spatial water maze task, but no significant neuropathology noted between the caffeine treated and the untreated groups.
(9)	HI at P6	1st dose of caffeine or saline directly post HI and 2nd dose of caffeine or saline at 24 h post HI	Male rats with HI treated with caffeine had less microglia activation relative to the saline-treated HI group, but the difference was not observed in the HI female rats.
(10)	HI from P3 to P12	Lactating dams containg either ccaffeine or water given from P3 to P12	Chronic caffeine treatment from P3 to P12 showed less disruption of myelination and reduced ventriculomegaly.
(11)	GM-IVH induced at P7	Caffeine or PBS given for 3 days consecutively post-lesion from P7 to P9	Caffeine reduced vascular damage and inflammatory markers, such as activated microglia.

P, post-natal; PBS, phosphate buffered saline; HI, hypoxia induced; GM-IVH, germinal matrix–intraventricular hemorrhage.

In this paper, we provide a summary of articles that we consider significant for understanding CC's neuroprotective role in neonatal cerebral pathologies. We also discuss the effects of CC on brain imaging and neurodevelopmental outcomes, examining published research on high vs. standard dosing protocols and the optimal timing of administration.

## Method

A search strategy with keyword search terms was built to identify articles pertaining to the effects of caffeine citrate on neurodevelopment in the last ten years. Our search strategy applied was: (caffeine) AND (neuroprotection) OR (caffeine) AND (neurodevelopment) OR (caffeine) AND (brain Magnetic resonance imaging) OR (caffeine) AND (cranial ultrasound). The online databases utilized include PubMed and EMBASE. We then selected articles published within the past 15 years that we think were relevant and aligned with our review's interest in elucidating molecular mechanisms of caffeine and its effects on neurodevelopment and neuroprotection in premature infants.

## Caffeine's neuroprotective effects and neurodevelopmental benefits in clinical studies

**Figure 1:**
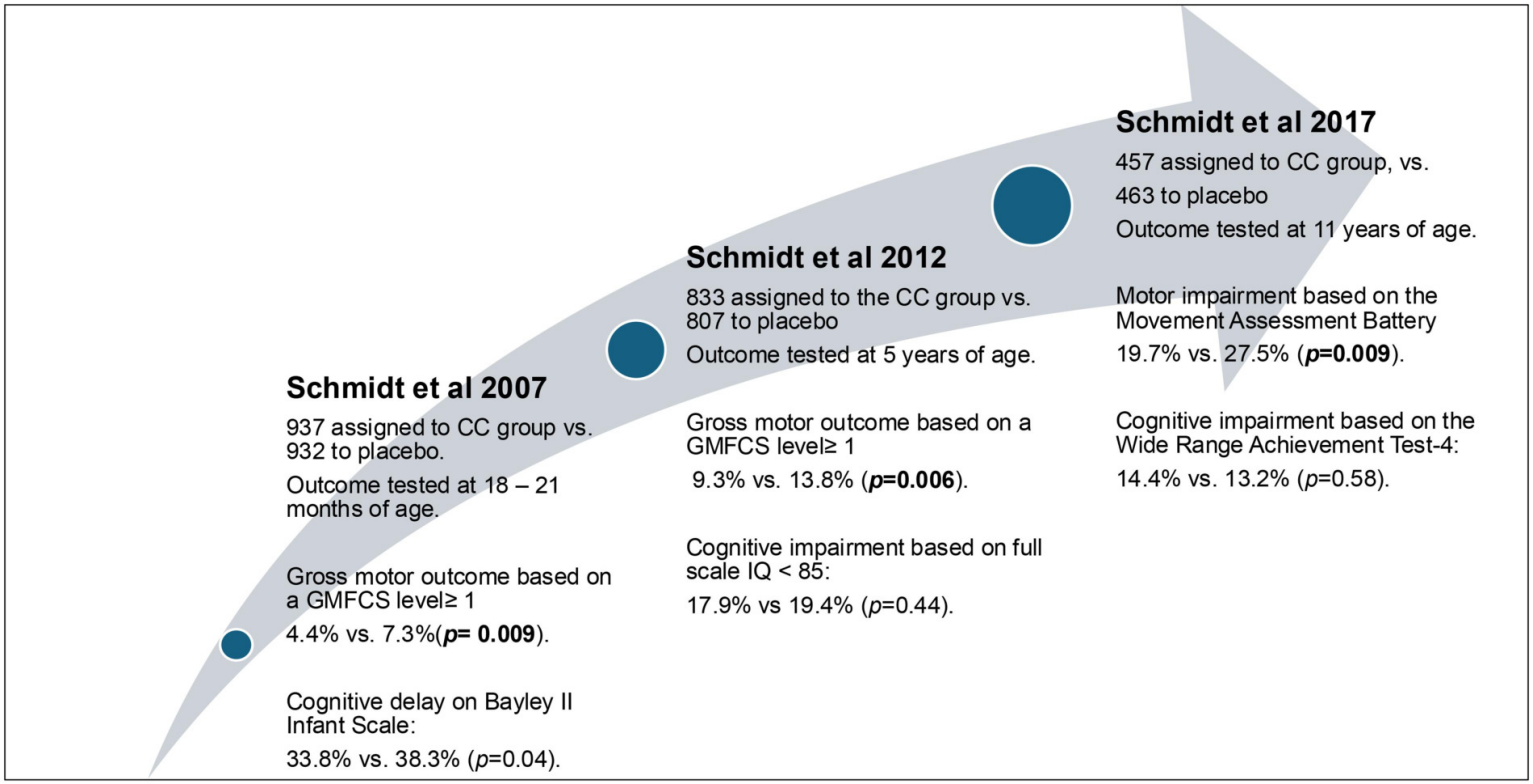
Caffeine citrate effect on neurodevelopmental outcome in the prospective studies. CC, caffeine citrate; GMFCS, Gross Motor Function Classification System; IQ, intelligence quotient.

CC's positive effect on neonatal neurodevelopment was first shown through the prospective Caffeine Therapy for Apnea of Prematurity (CAP) trial and its follow-up studies (Figure 1). The CAP trial published by Schmidt et al. in 2007 first showed that CC significantly reduces the risk of bronchopulmonary dysplasia (BPD) and incidences of cerebral palsy along with cognitive delay in preterm infants with low-birth-weight infants (32). Based on this study, CC has become part of the NICU protocol for BPD prevention and neuroprotection for premature infants born less than 32 weeks of gestation. The five-year follow-up of 96% of these participants of the CAP trial showed that the rates of death, motor impairment, and behavioral problems did not differ significantly between the two groups. The incidence of cognitive impairment was lower at five years than at 18 months and similar in the two groups, however the secondary analysis demonstrated improvement in gross motor function in the CC group (13). Another follow-up study investigated the specific relationship between CC therapy and developmental coordination disorder (DCD) at five years of age. The two groups of infants, those who received caffeine therapy and those who received a placebo, were assessed at five years of age using the Motor Assessment Battery for Children (MABC), clinical signs of cerebral palsy, and Full-Scale IQ. If a child's MABC was less than the 5th percentile, a Full-Scale IQ greater than 69, and did not have a diagnosis of cerebral palsy, the child was positive for DCD. The results indicated that the rate of DCD was lower in the group treated with CC (14).

To further investigate the long-term effects of CC therapy, a follow-up study was performed from 2011 to 2016 when the infants in the CAP trial reached 11 years of age. The study measured functional impairments through academic performance, motor skills, and behavior. Academic performance was assessed with the Wide Range Achievement Test-4; motor skills were assessed by the Movement Assessment Battery for Children, and behavioral problems were measured with the Child Behavior Checklist. The results indicated no differences in academic performance or behavioral problems; however, the CC-treated group had a significant reduction in risk of motor impairments (15). The CC-treated group performed better on fine motor coordination, visuomotor integration, visual perception, and visuospatial organization at 11 years of age (33).

The effect of CC on neuroimaging has been described in different studies with similar positive effects. As part of the CAP trial cohort, 199 participants from the Royal Women's Hospital in Melbourne underwent brain magnetic resonance imaging (MRI) without sedation at 38–42 weeks corrected age. These images were analyzed for qualitative white and gray matter abnormalities, quantitative cerebral volumes, and diffusion tensor imaging. The study showed that CC treatment had a positive effect on cerebral white matter diffusion and on the amount of mature white matter organization (14). Similar positive results on early brain MRI were shown by a study performed in China, which administered CC to infants at 20 mg/kg vs. placebo within 72 h after birth followed by a 10 mg/kg/day maintenance dose until 34 weeks corrected age. The study used diffusion tensor imaging and investigated fractional anisotropy (FA) and apparent diffusion coefficient (ADC). Increases in FA and decreases in ADC have been correlated with white matter development and maturation (16). Another study evaluated brain MRIs from infants at 39–45 weeks postmenstrual age in the greater Cincinnati area to evaluate diffuse white matter abnormalities (DWMA) on brain MRI. DWMA were quantified using a published algorithm. The results suggested that prolonged CC therapy reduced the risk for DWMA (34).

Current literature provides moderate evidence that a CC treatment course, in the early life of very premature infants, provides benefits in preventing bronchopulmonary dysplasia, but still provides low-certainty evidence to demonstrate long-term neurological benefits beyond the neonatal and infancy period (12). An improvement in gross and fine motor outcomes could be an important focus for future research.

## High vs. standard caffeine citrate dose administration

There is only one randomized controlled trial that investigated the effects of high vs. standard CC loading dose administration on brain imaging and neurodevelopmental outcomes. The study, performed between 2008 and 2010, included infants who were born at or under 30 weeks GA who were randomly assigned to receive either a high loading dose of CC of 80 mg/kg or a standard loading dose of 20 mg/kg. The study utilized brain MRI to assess the cranial structures between the two groups and neurodevelopmental outcomes at two years of age. The results showed an increased risk of cerebellar hemorrhage in preterm infants who received 80 mg/kg of CC as a loading dose. There was no difference in neurodevelopmental outcomes at two years of age using the Bayley III scale (17). This finding remained stable when 74% of the sample came back for follow-up at five years of age, with no significant differences in the neurodevelopmental scores between the two groups (18).

A retrospective study of 218 preterm infants who were followed over a 3-year period investigated the incidences of neonatal morbidities and neurodevelopmental outcomes when administered CC at a higher loading dose vs. a standard loading dose (80 mg/kg vs. 20 mg/kg) within their first 36 h of life. The decision to administer a higher vs. a standard loading dose was made by the neonatologists depending on clinical circumstances; these infants were typically of lower birthweight and had higher rates of mechanical ventilation. Instead of using MRI as the imaging modality, this study used cranial ultrasounds. The results showed no differences in the incidences of periventricular-intraventricular hemorrhage and cystic periventricular leukomalacia when using cranial ultrasound between the higher and standard doses. The researchers further noted that while there was no relationship noted between cerebral hemorrhage and high-loading doses of CC, there was an overall lower incidence of cerebral hemorrhage, which could be attributed to using cranial ultrasound imaging rather than MRI (19).

Regarding the maintenance dosing of CC after the loading dose, there is evidence that higher maintenance dosing may have an added neurodevelopmental benefit. Higher daily CC exposure over the NICU course was also associated with decreased odds of neurodevelopmental disability at 30 months CA, as shown by the Bayley III test for infant development. Motor, language, and cognitive composites were superior in neonates in the high daily dose group, despite an increased rate of BPD and lower GA in this same group (20).

Based on the current evidence, a loading dose of 20 mg/kg remains the standard, with caution against higher doses due to potential risks. The maintenance dose recommendation is still at 5 mg/kg/day, with potential benefit for higher maintenance dosing up to 10 mg/kg/day and beneficial cumulative CC exposure, especially in extremely premature infants.

## Timing of caffeine citrate administration

There is ongoing research regarding the time window of caffeine therapy initiation for maximal neuroprotection in preterm neonates. In hypoxia-ischemia murine models, a significant reduction in moderate to severe brain damage, along with partial functional recovery, was evident with immediate caffeine treatment, while its effect was limited at the late time points (21). In a prospective study of premature newborns of median 28-week GA, CC therapy initiated within the first 24 h of life was found to have a lower incidence rate of GM-IVH (41%) compared to the rate (57.6%) in the group that received CC on the second day of life or later (22). A retrospective study of preterm infants with less than 37 weeks GA from two hospitals in China also showed that GM-IVH incidence decreased in the group receiving early CC therapy (within 48 h of life) vs. the late group, in which CC was initiated over 48 h after birth (23). GM-IVHs were also observed at a higher rate in the late CC treatment group (24).

A recent meta-analysis published in 2023 reviewing 11 studies that investigated the timing of CC administration (25), two of them were prospective studies (26, 27). The analysis divided early (0–2 days) vs. late (> 3 days) CC initiation. In the group receiving early CC, there was a significant decrease in the rates of BPD, patent ductus arteriosus, GM-IVH, retinopathy of prematurity, and late-onset sepsis. The incidence of the composite outcome of BPD or death was significantly lower in the early CC-treated group. However, early CC administration was associated with a higher rate of mortality, which could be attributed to a survival bias. These results are in line with a prior meta-analysis done in 2015 showing the same results (28).

There are no prospective studies done on the timing of CC administration in premature infants in relation to neurodevelopmental outcomes. However, in retrospective studies involving 2108 neonates < 29 weeks GA, early administration of CC (within 2 days of birth) was found to be associated with less significant severe neurological injury on head ultrasound (parenchymal brain injury or ventriculomegaly with or without GM-IVH) and less significant neurodevelopmental impairment (defined as ≥1 of the following: cerebral palsy with gross motor function classification system levels III to V; Bayley-III cognitive, language, or motor score of <70; hearing aid or cochlear implant; or bilateral visual impairment) in the early CC-treated group (29). A smaller study in 2017 involving 160 very low birth weight newborns investigating the differences in outcomes between early (within 48 h of life) and late (> 48 h) CC administration showed that early CC administration was associated with significantly better composite cognitive, language, and motor outcomes on the Bayley III testing at 18–22 months CA (30). Interestingly, infants with high prenatal inflammatory risk have been found to have substantially better cognitive outcomes if treated with xanthine derivatives, including CC, within 48 h of birth, while no difference was seen in the low risk group with early (< 48 h) xanthine administration (31).

## Discussion

The available preclinical evidence has confirmed caffeine citrate's neuroprotective properties. Caffeine citrate's antioxidant ability and its antagonistic effects on adenosine receptors augment anti-neuroinflammation, prevent premature loss of oligodendrocyte progenitors in cerebral white matter, and maintain the integrity of the myelin sheath. There is strong clinical evidence to support that CC improves brain oxygenation due to improved respiratory drive and reduced ventilatory support, hence decreasing the risk of bronchopulmonary dysplasia in very premature infants. By improving respiratory status, along with the other neuroprotective properties, CC may have a positive effect on brain development.

Brain imaging studies via MRI have shown an improvement in white matter organization and density, as well as an indirect role in reducing white matter injuries in CC-treated neonates. Future studies should focus on white matter structural development through advanced MRI imaging (like diffusion tensor imaging and positron emission tomography) to assess the CC effect on brain inflammation and white matter recovery. This approach may be more informative than assessing the white matter injury itself, especially since CC may be playing a role in healing rather than preventing the injury. These imaging findings may or may not correlate with an improvement in neurodevelopmental outcomes, perhaps due to the limitations of the current imaging assessment or the confounding effects of post-discharge interventions like physical and occupational therapies, which could attenuate the long-term effect of CC. Focusing on more specific neurodevelopmental aspects as an outcome measure may better reveal the positive effects of CC, including on gross and fine motor development.

There is reasonable evidence supporting the early benefit of CC within 48 h after birth compared to a later time in prematurely born infants, especially regarding the occurrence of intraventricular hemorrhage and bronchopulmonary dysplasia. While this earlier administration may lead to further improvement of neurodevelopmental outcomes as well, further prospective studies are still required. The cumulative daily dose of CC, however, appears beneficial in enhancing cerebral maturation and development.
